# Ultrasound-guided anterior iliopsoas muscle space block compared with fascia iliaca compartment block in elderly hip surgery: A randomized controlled trial

**DOI:** 10.1097/MD.0000000000040521

**Published:** 2024-11-08

**Authors:** Emery Niyonkuru, Rui Zeng, Xu Zhang, Juan Zhu, Peng Jiang, Peng Ma

**Affiliations:** a Department of Anesthesiology, Affiliated Hospital of Jiangsu University, Zhenjiang, Jiangsu Province, China; b Jiangsu University, School of Medicine, Zhenjiang, Jiangsu Province, China.

**Keywords:** anterior iliopsoas muscle space block, hip fracture, supra-inguinal fascia iliaca compartment block

## Abstract

**Background:**

Elderly hip fracture patients experience severe pain. Surgical stress and pain during position changes for spinal anesthesia puncture can adversely affect hemodynamics. The objective of this study was to compare the perioperative analgesic efficacy of anterior iliopsoas muscle space block with supra-inguinal fascia iliaca compartment block (S-FICB) in elderly patients undergoing hip surgery.

**Method:**

In this randomized control trial, 66 patients were randomly assigned to either the iliopsoas space or the S-FICB group. Each patient received 30 mL of ropivacaine 0.375%. Perioperative pain scores were assessed and compared in both groups. The primary outcome was pain scores during changes in position for neuraxial anesthesia. Secondary outcomes included postoperative pain intensity, inflammatory markers for 12 to 24 hours, and perioperative adverse reactions.

**Results:**

The iliopsoas space group demonstrated a faster median block onset of 7 [6–8] minutes compared to S-FICB 14.50 [13–16] minutes (*P* < .001). Neuraxial anesthesia position changes resulted in lower pain scores for iliopsoas 2 [1–2] versus S-FICB 3 [3–4] (*P* < .001). Resting pain scores were similar at 12 hours post-surgery, but during exercise, the iliopsoas group had significantly lower scores [1–2] compared to S-FICB 4 [2–4] (*P* < .001). After 24 hours, C-RP levels were lower in the iliopsoas group (14.86 ± 1.23 mg/L) than S-FICB (17.90 ± 1.25 mg/L) (*P* < .001). The 2 groups differed from one another (*P* < .001). These findings suggest that iliopsoas space block may offer faster, superior dynamic pain control, and potentially reduced inflammation compared to FICB block for postoperative pain management.

**Conclusion:**

In elderly hip fracture patients, the anterior iliopsoas space block works slightly better than S-FICB at providing effective perioperative analgesia.

## 1. Introduction

Total hip arthroplasty (THA) has been one of the most significant advancements in orthopedic surgery over the past century. Given that hip fractures are typically associated with severe pain, effective analgesia is crucial both before and after surgery.^[1]^ Adequate pain management following THA helps to reduce complications and promotes postoperative mobilization.^[2]^ Continuous analgesia throughout the prehospital, preoperative, and postoperative phases, extending to final rehabilitation, is essential for the optimal care of patients with hip fractures.^[3]^ Various approaches are available to manage perioperative pain in elderly patients undergoing THA. These include injectable analgesia, epidural analgesia, local anesthesia infiltration protocols, and peripheral nerve blocks (PNBs). However, there is ongoing debate regarding the ideal analgesic method for this patient population.^[4]^ Opioids are powerful analgesics often used in THA, but their side effects such as sedation, nausea, vomiting, respiratory issues, and urinary retention pose significant risks, particularly in elderly patients.^[5]^

Regional nerve blocks, including lumbar plexus and quadratus lumborum blocks, are commonly used for postoperative pain relief following THA.^[6]^ Techniques such as femoral nerve blocks (FN) and fascia iliaca compartment blocks (FICB) have become popular for managing hip fracture pain and reducing opioid-related side effects, including delirium in elderly patients.^[7]^ More recently, the ultrasound-guided pericapsular nerve group block has emerged as an effective option, targeting the articular branches of the hip joint.^[7]^ While localized analgesia strategies are widely used to reduce opioid consumption the associated adverse effects have limitations.^[6,7]^ Local anesthetic infiltration into the surgical wound has gained popularity in THA and total knee arthroplasty due to its opioid-sparing effects.^[6]^ However, single-shot PNBs are challenging in THA due to the complex innervation involved.^[8]^

The supra-inguinal FICB (S-FICB) provides a more comprehensive sensory block than the infra-inguinal FICB (I-FICB) approach, covering the medial, anterior, and lateral regions of the thigh. The S-FICB also offers a more consistent spread of local anesthetic under the fascia iliaca and around the psoas muscle.^[8]^ However, studies show mixed results regarding its efficacy. For instance, some trials found that FICB did not improve pain, narcotic use, or function in patients undergoing THA via the mini-posterior approach but did cause increased quadriceps weakness.^[9]^ The effectiveness of these blocks depends on adequately covering the hip joint’s articular branches, which arise higher along the nerves.^[10]^ FICB is increasingly used for hip fracture analgesia, offering efficient postoperative pain control with minimal adverse effects.^[11]^ However, magnetic resonance imaging (MRI) studies have shown that the local anesthetic spread following FICB does not consistently cover the obturator nerve (ON), leading to incomplete analgesia.^[10]^ In contrast, spinal anesthesia (SA) has gained popularity in difficult patient populations, such as those undergoing revision THA.^[12]^ Despite its advantages, achieving proper patient positioning during SA can be challenging due to pain and the need to stabilize the limb.^[13]^ FICB, when administered before SA, has been shown to reduce positioning pain and shorten the time required for SA puncture.^[14]^

The fascia iliaca compartment encompasses the FN, lateral femoral cutaneous nerve (LFCN), ON, and genitofemoral nerve (GFN), all of which lie within the same fascial membrane.^[15,16]^ An injection of local anesthetic into this compartment achieves a success rate of 67% to 90% for anesthesia of the hip, knee, and thigh.^[17]^ However, the success of S-FICB depends on large volumes of local anesthetic, and its efficacy is often limited without this optimal distribution.^[18]^ The hip joint is innervated by both the lumbar plexus (femoral and ONs) and the sacral plexus (sciatic nerve, superior gluteal, and quadratus femoris nerves). Blocking the lumbar plexus can paralyze the anterior hip capsule, which contains the majority of sensory nerve endings.^[19]^ However, lumbar plexus blocks carry risks such as epidural spread, psoas abscess, retroperitoneal hematoma, and systemic toxicity from local anesthetics.^[20]^

Previous studies have suggested that blocking the iliopsoas space may be as effective as traditional lumbosacral plexus blocks.^[21]^ The iliopsoas space block targets the lumbosacral trunk as it passes beneath the psoas major muscle, offering potential advantages over the traditional sacral plexus block, which is performed in the supine position.^[21]^ In comparison, the iliopsoas block (IPB) has been shown to improve quadriceps strength and shorten the time to ambulation post-THA without significantly affecting pain scores, total opioid consumption, or patient satisfaction.^[22,23]^ Although there is growing interest in the use of the IPB, no studies have yet compared its efficacy to that of S-FICB for perioperative analgesia in hip surgery. Our study aims to determine whether the anterior iliopsoas space block provides superior perioperative analgesia compared to the S-FICB in patients undergoing hip surgery. The primary outcome measure is pain during spinal anesthesia positioning, while secondary outcomes include postoperative pain intensity, inflammatory markers 12 to 24 hours post-surgery, and perioperative adverse events.

## 2. Method

### 2.1. Ethics and trial registration

The trial protocol is under the Declaration of Helsinki. This research was approved by the Institutional Ethics Committee of the Affiliated Hospital of Jiangsu University before the start of the study (the approval number for this study is KY2022H1209-2). This study was registered in the Chinese Clinical Trial Registry on December 17, 2022 (ID: ChiCTR2200066797). Each patient who was enrolled in the study had provided informed consent before participating in the study.

### 2.2. Patients

In this study, 66 elderly patients with hip fractures aged 60 to 80 years with an American Society of Anesthesiology II to III classification were enrolled. We included all patients who met the inclusion criteria and were scheduled to undergo hip surgery under spinal anesthesia between January 2023 and September 2023. Exclusion criteria were coagulation disorders, local anesthetic allergies, neurological deficiency in a lower limb, sepsis at the puncture site, drug/alcohol dependence, chronic pain reliever usage, associated with severe organ dysfunction such as poor function of the heart, liver, or kidney, or inability to cooperate, block failure, previous cognitive function disorders like dementia or delirium, schizophrenia, Parkinson disease.

### 2.3. Randomization and preoperative assessments

Patients were assigned randomly to either the anterior iliopsoas muscle space block or the S-FICB group using a computer-generated randomization table. In both groups, they underwent preoperative examinations including blood tests, kidney function, blood type, glucose levels, electrocardiogram (ECG), and chest X-rays, cognitive function performance. All patients fasted 8 hours before surgery. Patients were prepared in the preoperative room after confirming fasting status for 8 hours and undergoing a brief review examination. Vital signs and ECG were monitored, and intravenous access was established. Patients were given ringer’s lactate via peripheral intravenous access.

### 2.4. Interventions and outcomes

We have performed routine disinfection before every single procedure that we have performed. Betadine was applied to the skin surrounding the block space and covered with window protective coverings. Sterilized covers were used to cover the probe. One experienced anesthesiologist performed ultrasound-guided nerve blocks using a 22-G needle, a 2- to 5-MHz curved array transducer, and a mobile ultrasound device (SonoSite, M-Turbo, Bothell, WA). Local anesthesia was administered with ultrasound guidance, and the effectiveness of the nerve blocks was assessed. Pain was evaluated using a simple verbal scale of 0 to 4 after the procedure in the preoperative room. All patients who experienced nerve block failures or spinal anesthetic failure after surgery or who were lost to follow-up following surgery as a result of being admitted to the intensive care unit were excluded from the study.

### 2.5. Supra-inguinal fascia iliaca compartment block

**Figure 1.**
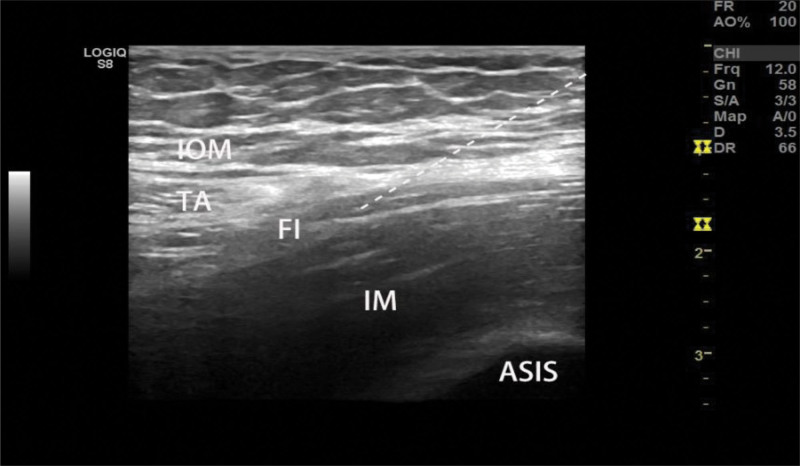
Ultrasonic image of supra-inguinal fascia iliaca compartment block. ASIS = anterior superior iliac spine, FI = fascia iliaca, IM = iliac muscle, IOM = internal oblique muscle, TA = transversus abdominis.

As in previous studies, FICB was carried out in the preoperative room while the patient was lying supine under ultrasound guidance.^[24]^ Over the inguinal ligament, the probe was positioned. The femoral artery and FN were identified, and the side of the FN became a focal point on the surface of the screen. To photograph the parasagittal plane, a probe was subsequently turned cranially. Using the deep circumflex iliac artery as a guide, the needle was inserted superficially into the fascia iliaca and approximately 2 cm ahead of the ligament of the inguinal cavity. By inserting the needle from caudal to cranial until its tip was beneath the fascia iliaca, the deep circumflex iliac artery could be successfully accessed. Then, following a single injection of 1 to 1.5 mL of local anesthetic to make sure that the fascia iliaca and iliaca muscle had been correctly hydro-dissected, 30 mL of 0.375% ropivacaine was administered in different aliquots. The iliac fossa and fascia iliaca were both deeply injected with local anesthetic to help it reach the proximal lumbar plexus. The needle was passed cranially between injections, only entering the gap left by the distending fluid (Fig. 1). By moving the probe in parallel to the inguinal ligament during injection, it was possible to confirm that the local anesthetic had been evenly distributed around the FN in the short-axis view.^[19]^

### 2.6. Anterior iliopsoas space block

**Figure 2.**
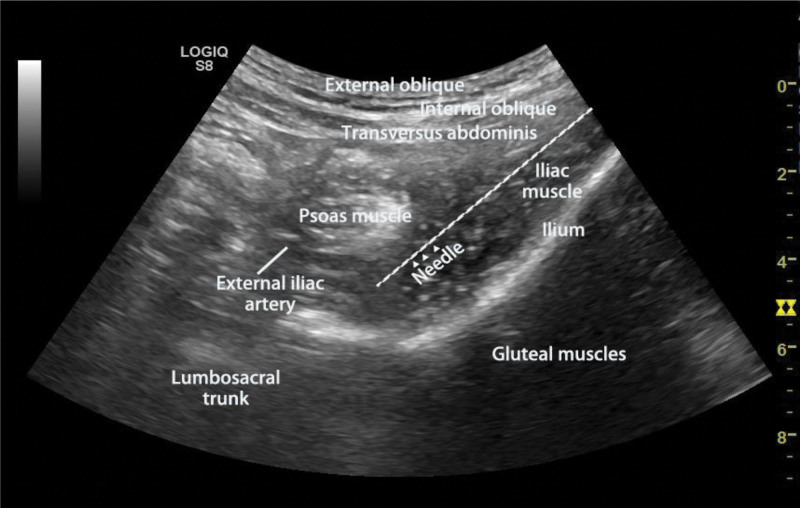
Ultrasonic image of anterior iliopsoas space block. EIA = external iliac artery, IM = iliac muscle, PM = psoas muscle.

The patients in this group were lying on their backs, and in the transverse section of the ultrasound scan, the lowest speed, 2 to 5 MHz, curving array transducer was utilized. It was positioned 3 to 4 cm medial to the anterior portion of the superior iliac spine. LFCN develops onto the side aspect of the psoas major muscle and then navigates through the fascia iliaca at the anterior superior iliac spine before traveling obliquely over the anterior face of the iliac muscle. The FN travels through the back of the psoas muscle and fascia iliaca at the slant formed by iliac muscles and psoas major. The ON spirals via the back of the psoas major muscle. The psoas major muscle is in the medial part of the lumbosacral trunk. Blocking ON and FN, a needle was inserted anteriorly to posteriorly through the iliac muscle and pushed inwardly to the injection terminus into the iliopsoas space (Fig. 2). Local anesthetics which spread the back of the psoas major muscle can block LFCN. The injection endpoint, which is situated in the iliopsoas space was attained by advancing the needle in-plane. A volume of 30 mL of 0.375% ropivacaine was administrated into the iliopsoas muscle.^[20]^ The blocks were assessed by a medical professional who didn’t participate in the region block’s administration.^[25]^ The pinprick test was used to evaluate sensory blocks in lower limbs. Through thermal imaging and infrared cameras, the effect and extent of the nerve blockages were evaluated and recorded. We evaluated the skin’s temperature of the bilateral lower body parts 30 minutes post the completion of the nerve block following previous research on the onset time of nerve blocks^[21]^

### 2.7. Intraoperative management

Subarachnoid space block anesthesia was performed for all patients. After positioning the patients laterally with the afflicted leg above, skin disinfection was done followed by local infiltration with lidocaine 1%. A thin needle (25 G) was inserted into the lumbar spinal space between lumbar vertebrae L3 to L4 and 2 mL of ropivacaine 0.5% was administered into the subarachnoid space. Vascular fillings were performed using intravenous infusion of ringer lactate solution or sodium chloride.^[26]^

### 2.8. Intraoperative recordings and effects management

Intraoperative patients’ vital indicators were continuously monitored during surgery, particularly blood pressure, pulse, ECG, and oxygen saturation. Conditions such as low BP, arrhythmia, hypoxemia, headache, pain, nausea, and vomiting were noted. Treatment included phenylephrine or epinephrine for hypotension, IV atropine for bradycardia, and IV ondansetron for nausea and vomiting. Oxygen was administered to prevent hypoxia, and parecoxib sodium was used for inadequate analgesia.^[27,28]^

### 2.9. Postanesthesia care management

As in previous studies, after hip surgery, all patients were transferred to the postoperative room and endpoints were measured in the postoperative room by a blinded researcher. The outcomes were assessments of postoperative pain in 12 and 24 hours after surgery. Pain was assessed using visual analogue scale (VAS). Along with probable spinal anesthesia complications (nausea, vomiting, and urine retention), the rate of adverse events has been documented. Bedside monitoring was accomplished on postoperative day 1 to day 3, by a blinded investigator. Undesirable block-related events and VAS pain scores were noted. C-reactive protein (CRP) and interleukin-6 (IL-6) levels were measured 24 hours after surgery.

### 2.10. Statistics analysis

The pain scores when switching posture for neuraxial anesthesia were the key finding. Before surgery, we hypothesized that the anterior iliopsoas muscle space block would perform better than S-FICB. Given that the objective of the research was to verify superiority equivalency, the population standard deviation in both groups was predicted to be σ = 0.6, the effectiveness cutoff MS to be 1.1, and the difference in pain scores in both groups to be =1.6. With an alpha of 2.5% and a minimum statistical power of 80%, we computed those 26 patients in each group would be required to be registered using PASS15.0. We intended to enroll a total of 66 patients (33 patients in each group), which would account for a 20% drop-out and protocol violation rate.

Measurements and enumeration data are the primary components of data analyses. Age, body mass index, the length of the surgery, the VAS, the onset of surgery, hematology-related values, and the degree of inflammatory markers were all included in the measurement data. Gender, ASA classification, surgical modalities, and adverse effects were all included in the enumeration data. The Shapiro–Wilk test was performed to determine if the measurement data was normally distributed, X ± S was employed to display the distribution, and an independent sample *t* test was utilized to compare 2 groups, with the Chi-square test. A value of *P* < .05 based on a two-tailed probability was regarded as statistically significant for all statistical analyses, which have been carried out by SPSS 27.0.

## 3. Results

**Figure 3.**
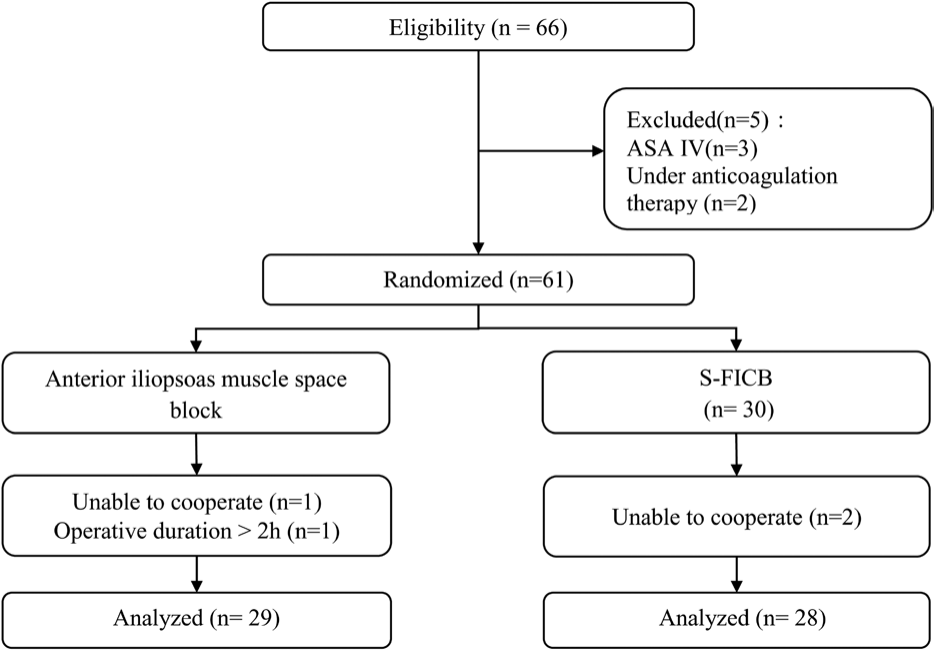
Diagram showing the flow of the unified standards of reporting trial statement. This diagram depicts the flow of participants through the study, in accordance with the Consolidated Standards of Reporting Trials (CONSORT) guidelines.

While 66 participants had their eligibility evaluated, 5 of them were found to be disqualified due to eligibility requirements or patient disapproval; as a result, 61 victims were taken into consideration overall. Four patients were excluded because 3 were unable to cooperate with researchers and 1 patient in the anterior iliopsoas muscle space block group was excluded from the analysis because of the surgery time of >2 hours (Fig. 3). The analysis’s ultimate sample size was 57 patients: 29 within the anterior iliopsoas muscle space group while 28 within the S-FICB. Both the baseline parameters and the participant’s demographic characteristics were equally allocated among the randomized groups (Table 1). The type and duration of surgery were also similar between the groups.

**Table 1:** Baseline and demographic data.

Characteristics	Anterior iliopsoas muscle space block (n = 29)	S-FICB (n = 28)	*P*-value
Sex (M/F)	13/16	11/17	.672
Age (years)	74.72 ± 4.60	75.29 ± 5.02	.661
*ASA*			
I	1	2	.716
II	26	25	
III	2	1	
BMI (kg/m^2^)	22.29 ± 0.70	22.29 ± 0.71	.934
Surgical modalities			
Total hip replacement	21	20	.934
PFNA	8	8	
Duration of surgery (minutes)	56.59 ± 6.95	56.21 ± 8.48	.857

Values are numbers or mean ± SD. The mean ± SD is used to express data.

BMI = body mass index, PFNA = proximal femoral nail antirotation, SD = standard deviation, S-FICB = supra-inguinal fascia iliaca compartment block.

### 3.1. Perioperative pain assessment

**Figure 4.**
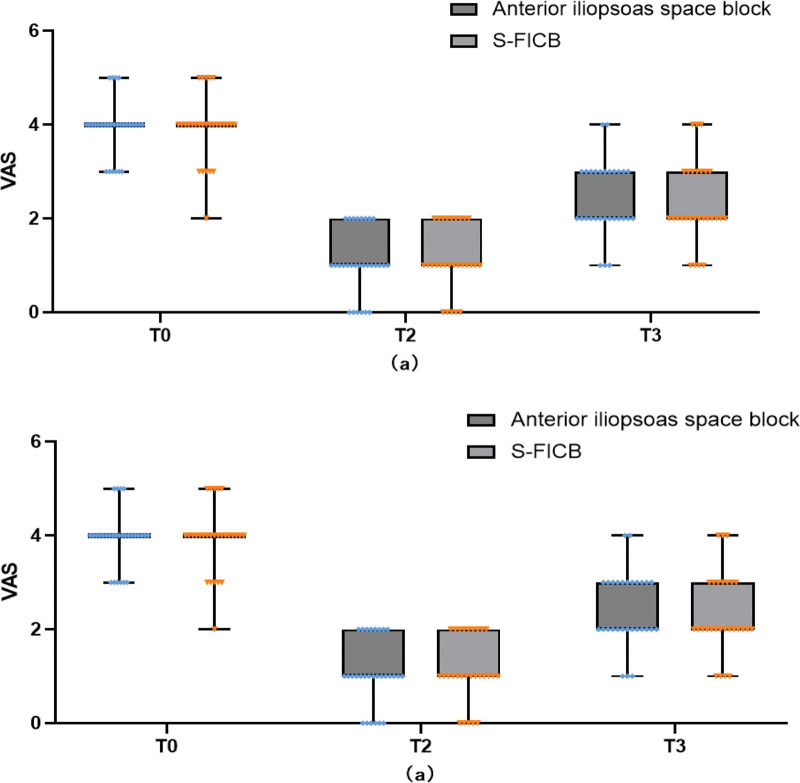
Effect of either an anterior iliopsoas space or S-FICB on postoperative pain. This figure compares postoperative pain scores between the anterior iliopsoas space block and S-FICB groups, measured using the Visual Analog Scale (VAS) from 0 (no pain) to 6 (severe pain). (A) Pain at rest; (B) pain during movement. Time points: T0 (preoperative), T1 (neuraxial anesthesia in positioning), T2 (12 hours post-surgery), and T3 (24 hours post-surgery).

Different time points were used to assess perioperative pain and analgesia satisfaction (Table 2). Pain scores when changing positions for neuraxial anesthesia in the iliopsoas space group were 2 [1–2] and 3 [3–4] in the S-FICB group (*P* < .001). The difference in pain scores in all groups was not noticeable at rest 12 hours after surgery (Fig. 4A), but when the patient exercised, the pain score 1 [1–2] in the anterior iliopsoas muscle space groups was significantly <4 [2–4] into S-FICB group (*P* < .001) (Fig. 4B). Postoperatively, when the patient develops sharp pinprick pain at the surgical site, an additional pain assessment is performed. Once the pain score is >4, parecoxib sodium 40 mg is given for salvage analgesia, repeated if necessary. The number of required additional analgesia in the iliopsoas space group were 3 and, in the S-FICB group were 2 (10.3% vs 7.14%, *P* = .669).

**Table 2:** VAS score comparison.

	Anterior iliopsoas muscle space block (n = 29)	S-FICB (n = 28)	*P*-value
*VAS*			
Preoperative resting state	4 (0)	4 (0)	.889
Preoperative exercise state	8 [7–8]	8 [7 to 8]	.986
Neuraxial anesthesia in positioning	2 [1–2]	3 [3 to 4]	<.001*
Rest state 12 hours after surgery	1 [1–2]	1 [1 to 2]	.688
Exercise state 12 hours after surgery	1 [1–2]	4 [2 to 4]	<.001*
Rest state 24 hours after surgery	2 [2–3]	2 [2 to 3]	.578
Exercise state 24 hours after surgery	4 (0)	4 (0)	.568

Data are expressed as median (IQR).

S-FICB = supra-inguinal fascia iliaca compartment block, VAS = Visual Analogue Scale.

* *P* < .05.

### 3.2. Onset and operation time of nerve block

**Figure 5.**
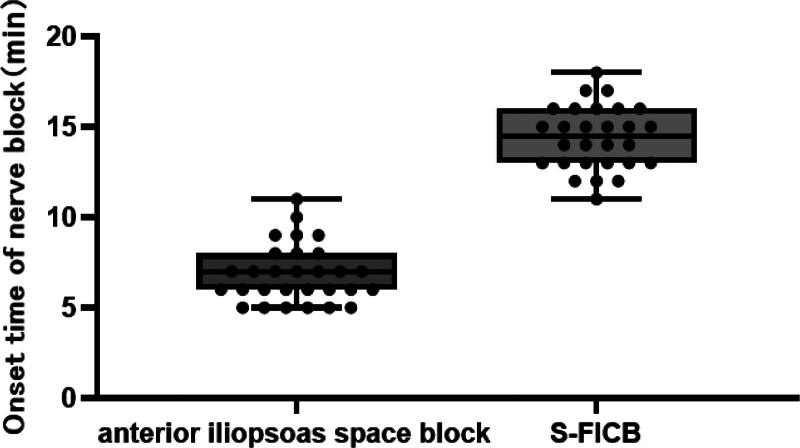
Comparison of onset time of nerve block. This figure compares the onset time of nerve block between 2 groups: anterior iliopsoas space block and S-FICB. The onset time is measured in minutes.

**Figure 6.**
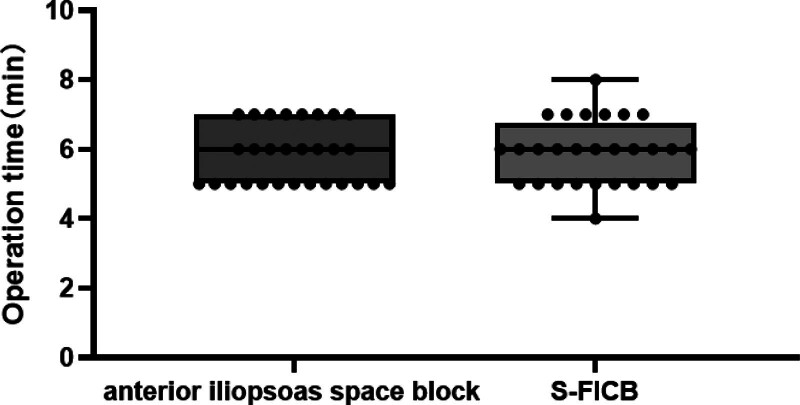
Comparison of operation time of nerve block. This figure compares the operation time between 2 groups: anterior iliopsoas space block and S-FICB. The operation time is measured in minutes.

In hip surgery, anterior iliopsoas space block and S-FICB are effective perioperative pain relievers. However, the start time of the anterior iliopsoas muscle block was faster 7 [6–8] minutes than in the FICB 14.5 [13–16] minutes (Fig. 5). Nonetheless, there is no difference in operation time, which is approximately 6 minutes (Fig. 6).

### 3.3. The blocking range of the iliopsoas space block

After the application of the nerve block, thermal imaging with the FLIR infrared camera indicated that the skin’s temperature in the zone innervated by the sciatic nerve, FN, ON, and LFCN on the blocked space was considerably greater than it was on the unblocked side. The pinprick test confirmed that the LFCN, FN, and ON were correctly blocked.

### 3.4. Inflammatory

**Figure 7.**
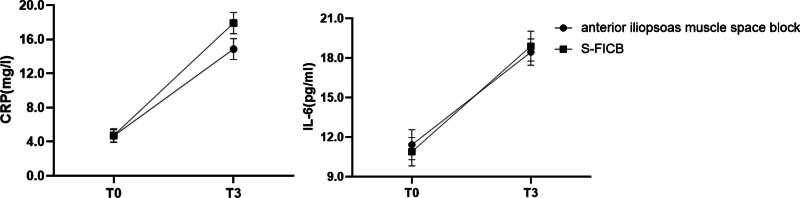
Comparison of CRP and IL-6. This figure compares the levels of C-reactive protein (CRP) and interleukin-6 (IL-6) between 2 groups: anterior iliopsoas muscle space block and S-FICB. The levels are measured at 2 time points: T0: preoperative; T3: 24 hours after surgery. FICB = fascia iliaca compartment block.

At 24 hours after surgery, CRP and IL-6 levels were measured to assess the inflammation (Table 3). CRP in the anterior iliopsoas muscle space group was 14.86 ± 1.23 mg/L and 17.90 ± 1.25 mg/L in the S-FICB group. The 2 groups differed from one another (*P* < .001) (Fig. 7).

**Table 3:** Comparison of CRP and IL-6.

	Anterior iliopsoas muscle space block (n = 29)	S-FICB (n = 28)	*P*-value
CRP (mg/L) T0	4.67 ± 0.73	4.72 ± 0.81	.798
CRP (mg/L) T3	14.86 ± 1.23	17.90 ± 1.25	<.001*
IL-6 (pg/mL) T0	11.42 ± 1.14	10.89 ± 1.07	.079
IL-6 (pg/mL) T3	18.45 ± 1.00	18.90 ± 1.13	.116

The mean ± SD is used to express data.

CRP = C-reactive protein, IL-6 = interleukin-6, SD = standard deviation, S-FICB = supra-inguinal fascia iliaca compartment block, T0 = before surgery, T3 = 24 hours after surgery.

* *P* < .05.

No differences in nausea, vomiting, pruritus, infection, or hypertension across groups were observed to be statistically significant (Table 4). The use of ropivacaine has not caused any adverse effects. Both groups did not exhibit any instances of vascular puncture or paresthesia.

**Table 4:** Comparison of adverse reactions.

Complications, n (%)	Anterior iliopsoas muscle space block (n = 29)	S-FICB (n = 28)	*P*-value
Nausea	3 (10.34)	2 (7.14)	.669
Vomiting	2 (6.90)	3 (10.71)	.610
Pruritus	0 (0)	1 (3.57)	.491
Infection	1 (3.45)	0 (0)	.322
Hypertension	3 (10.34)	3 (10.71)	.964

Values are numbers (%).

S-FICB = supra-inguinal fascia iliaca compartment block.

## 4. Discussion

The findings of this study demonstrate a notable difference in pain scores between the 2 groups during the transition of positions under neuraxial anesthesia and physical activity post-surgery, with the anterior iliopsoas space block group exhibiting superior pain control (*P* < .001). The group that received the anterior iliopsoas space block had better pain control compared to S-FICB, indicating that the iliopsoas space block may be more effective in managing pain and preventing hemodynamic fluctuations related to position changes. In secondary outcomes, we compared the impact of 2 different methods on postoperative pain and the systemic inflammatory response. We found no significant differences in pain scores among all groups 12 hours after surgery at rest (Fig. 4A). However, during physical movement, the pain score in the anterior iliopsoas muscle space group was notably lower (1 [1–2]) than in the S-FICB group (4 [2–4]) (*P* < .001). Additionally, levels of CRP and IL-6 in the blood 24 hours after surgery suggest that the anterior iliopsoas space block effectively reduced the release of inflammatory markers after surgery [14.86 ± 1.23 mg/L vs 17.90 ± 1.25 mg/L] (*P* < .001) (Fig. 7). These findings suggest that the anterior iliopsoas space block has the potential to mitigate perioperative pain, the surgical stress response, and support recovery.

The complicated innervation of the hip joint, supplied by numerous nerves, poses a significant challenge for effective analgesia and regional anesthesia during hip arthroplasty procedures.^[29]^ Compared to other anterior iliopsoas block methods, such as the FNB or the S-FICB, the anterior iliopsoas space block has demonstrated superior blocking quality. This single-shot technique targets a wider range of nerves.^[20,21]^ By effectively blocking the lumbosacral trunk, this approach significantly reduces pain and hemodynamic fluctuations associated with position changes, which is crucial for enhancing recovery in the perioperative period.^[21]^ The anterior iliopsoas space block is administered in the outermost part of the anterior iliopsoas space, allowing for proper distribution of the local anesthetic to provide proportional analgesia without exceeding its limits.^[21]^ In contrast, studies suggest that the S-FICB technique may not adequately reach the ON, as it is situated medially and posteriorly to the psoas muscle and disconnected from the lesser pelvic compartment.^[19,30]^ This difference in the spread and distribution of the local anesthetic may contribute to the superior analgesic effects and anti-inflammatory properties observed with the anterior iliopsoas space block. The management of pain in elderly patients undergoing hip surgery remains a significant challenge, as they often face various preoperative and postoperative complications, including immobility, increased risk of opioid-related adverse effects, and inflammatory responses.^[4,31]^ The prevention of postoperative pain is a crucial component of the rehabilitation process, as it can impact surgical stress, inflammation, and overall recovery.^[32]^ Recent studies suggest that the anterior iliopsoas space block may provide better analgesia compared to other techniques, such as S-FICB and lumbar plexus block, potentially allowing for quicker hospital discharge, and improved functional outcomes.^[20,21]^

Surgical trauma has been associated with a systemic inflammatory response, which has been implicated in the development of postoperative complications, such as delirium and cognitive decline.^[33–35]^ The reduction in the release of inflammatory markers, such as CRP and IL-6, observed with the anterior iliopsoas space block within 12 hours after surgery is particularly noteworthy. By effectively attenuating this inflammatory response, the anterior iliopsoas space block may mitigate the risk of these postoperative complications, potentially improving overall recovery and outcomes in elderly patients undergoing hip surgery. These results are supported by existing literature, which has shown the advantages of regional anesthesia techniques, such as PNBs like S-FICB. These techniques help improve pain management, decrease opioid use, and enhance outcomes for elderly patients undergoing hip fracture repair and arthroplasty.^[36–39]^ For instance, a systematic review by Macfarlane et al found that the use of regional anesthesia, such as PNBs, was associated with improved pain control, reduced opioid consumption, and faster functional recovery compared to general anesthesia in patients undergoing THA.^[40]^ Furthermore, the study highlighted the effectiveness of PNB techniques, including FICB, which are advantageous for THA patients because they provide sufficient analgesic effects postoperatively, particularly during the first postoperative period of resting discomfort, without weakening the quadriceps.^[41]^ Suitable anesthetic and analgesic techniques can prevent postoperative complications, improve pain control, and accelerate recovery.^[42]^ Additionally, the combination of regional anesthesia techniques with opioids/non-opioids enhances analgesic efficacy, reduces opioid side effects, and supports rehabilitation programs, leading to better outcomes and potentially lower costs.^[42]^ FICB has been widely shown to successfully reduce postoperative pain in patients undergoing hip surgery, such as THA. Studies demonstrate that FICB significantly reduces pain, decreases opioid consumption, shortens rehabilitation time, and improves cognitive function, with a more favorable safety profile compared to other regional blocks.^[43]^

While the use of single-injection PNBs has been the focus of previous studies, findings from this investigation suggest that a more targeted approach, such as the anterior iliopsoas space block, may provide superior analgesia and anti-inflammatory effects compared to the S-FICB. This may be due to the block’s potential ability to effectively target the lumbosacral trunk, which is believed to play a crucial role in innervating the hip joint.^[20]^ The management of pain with opioids is frequently combined with other multimodal therapies to optimize results and attain sustained pain relief in the long term. However, elderly patients are particularly vulnerable to the negative effects of opioids, such as respiratory depression, due to altered pharmacodynamics and underlying medical conditions.^[36]^ The use of regional analgesics, such as the anterior iliopsoas space block, may assist in reducing opioid abuse and its associated adverse effects. This is supported by a study by Schulte et al, which demonstrated that individuals receiving a FICB before surgery significantly reduced morphine milligram equivalents consumed following the procedure compared to those who did not receive the block.^[38]^

Previous research on single-shot PNBs for hip surgery highlighted risks such as nerve injury, local anesthetic overdose, toxicity, and excessive anesthetic concentration with blocks like FICB and lumbar plexus.^[4]^ Elderly patients undergoing hip arthroplasty often have reduced multi-system function and comorbidities. To minimize the risk of toxic reactions and complications, it is safer to use the minimum effective volume of local anesthetics.^[25]^ The FICB is a low-risk, effective pain relief technique that does not require surgical training, demonstrating higher success rates in blocking the femoral, lateral cutaneous, and ONs compared to the FN or “three-in-one block,” making it ideal for prehospital analgesia.^[44]^ However, a pilot study determined that a 40 mL injection volume can effectively target the femoral, obturator, and lateral femoral cutaneous nerves using a single-injection, ultrasound-guided S-FICB.^[45]^ In contrast, MRI findings have indicated that the spread of local anesthetic following the FICB does not cover the ON, suggesting this block may not provide effective analgesia.^[7]^ However, the study by Kris Vermeylen et al shows that the S-FICB with 40 mL of local anesthetic more reliably spreads the anesthetic to the anatomical location of the 3 target nerves of the lumbar plexus on MRI than the inguinal FICB.^[8]^ Nonetheless, 2 MRI studies on S-FICB using 30 to 40 mL showed the injected solution failed to reach the ON, retropsoas, or retroperitoneal cavity due to insufficient medial spread.^[30]^ The use of high volumes of local anesthetics, such as 40 to 60 mL for FICB, can lead to a higher incidence of local anesthetic systemic toxicity (LAST) and postoperative complications like falls due to muscle weakness. This was evident in studies by Allegri et al, where individuals experienced LAST and fall after receiving large doses of local anesthetics.^[14]^

The study found that the effective volumes (EV) of 0.25% ropivacaine for ultrasound-guided S-FICB in 21 patients with hip fractures were 15.01 mL for the EV50 and 26.99 mL for the EV95, using logistic regression analysis.^[46]^ However, the recommended concentration range for PNB is 0.3% to 0.5% ropivacaine. A lower concentration of 0.33% ropivacaine was chosen for elderly patients, who are more sensitive to local anesthetics.^[25]^ The blocking effect of other ropivacaine concentrations in different populations requires further study. The efficacy of the ON block in analgesia after hip arthroplasty remains uncertain.^[25]^ The EV95 offers effective pain relief during surgery while using a lower dose of local anesthetic, thereby reducing the risk of inadequate or ineffective pain management due to insufficient anesthetic volume.^[25]^ Zhang et al determined that the 95% EV (EV95) of 0.33% ropivacaine for ultrasound-guided S-FICB was 34.06 mL, providing effective analgesia for hip arthroplasty while minimizing local anesthetic use.^[25]^ However, elderly patients with reduced organ blood flow and liver function have decreased clearance of local anesthetics, increasing the risk of local anesthetic systemic toxicity. Additionally, patients with severe kidney, liver, or heart disease are especially vulnerable due to impaired clearance and reduced tolerance to physiological changes.^[47]^ High doses of local anesthetics raise the risk of toxicity, especially in lean patients with hip fractures. Careful consideration of the volume used in regional blocks is essential to minimize the risk of local anesthetic toxicity.^[3]^

A case report describes a 74-year-old patient who experienced LAST on 2 occasions following total knee replacement surgeries. The patient exhibited unusual symptoms, including respiratory difficulties and an altered mental state, underscoring the potential risks associated with these procedures.^[48]^ Rauf Gül and colleagues concluded that infra-inguinal FICB with 0.5 mL/kg of local anesthetic is a safe and effective method for postoperative pain relief after femur and knee surgery.^[49]^ The volume of local anesthetic used directly affects the duration of analgesia, with 0.5 mL/kg providing the best results. This block can reduce the need for additional analgesia and improve overall pain management.^[49]^ In our trial, we administered 30 mL of ropivacaine at a concentration of 0.375% to mitigate the risk of inadequate analgesia and LAST, in accordance with the standard protocols established at our institution. However, we did not specifically assess the minimum effective volume and concentration required for optimal analgesic outcomes in the anterior block. Consequently, further investigations are warranted to elucidate the ideal volume and dosage of ropivacaine for the anterior iliopsoas space block in the elderly population, as well as to evaluate the duration of its analgesic efficacy.

Older individuals experiencing hip fractures face significant preoperative challenges, including the need for effective postoperative pain management and reducing reliance on opioid medications during surgical procedures.^[4]^ Inadequate pain control in this population can lead to serious health complications associated with immobility, such as atelectasis, myocardial infarction, increased cardiac workload, deep vein thrombosis, pulmonary emboli, and pneumonia.^[31]^ Additionally, the severity of postoperative pain is an important factor, as it can promote the release and activation of inflammatory cytokines, which may, in turn, accelerate the development of delirium.^[33]^ These findings are supported by existing literature, which has demonstrated the correlation between postoperative inflammation and the development of postoperative delirium and cognitive dysfunction.^[34,35]^ A study by Kragsbjerg et al indicates that serum CRP rises shortly after surgery, increases 48 to 96 hours later, and then declines a little over 3 weeks later. Proinflammatory cytokines such as IL-1, TNFα, and IL-6 are generated more when inflammation occurs, along with fibrinogen, another acute-phase reactant.^[50]^ According to the study, there is a strong correlation between acute postoperative discomfort and delirium since it stimulates pro-inflammatory mediators to be released more quickly, which in turn accelerates the development of delirium. By preventing the release of cytokines that trigger inflammation, the anterior iliopsoas space block’s anticipatory analgesic effect can help avoid perioperative delirium. Furthermore, cutting back on opioid use can also lessen the production of cytokines that promote inflammation.^[33]^ Additional research is needed to assess the impact of the anterior iliopsoas space block on postoperative delirium, venous thrombosis, and other related complications. A thorough understanding of these associations could improve patient outcomes in the postoperative period.

In a double-blind prospective study, 80 primary THA patients received either an anterior iliopsoas space block with local infiltration analgesia. The anterior space block group used less morphine, had lower pain scores, and recovered faster, without affecting quadriceps strength or complications.^[51]^ It has recently been demonstrated that surgical trauma-related peripheral inflammation and the subsequent release of systemic inflammatory markers impact central nervous system inflammatory mechanisms by activating neurogliocytes, leading to the simultaneous endogenous production of pro-inflammatory cytokines.^[52]^ Acute inflammation phase response, neutrophil leukocytosis, lymphocyte development, and cytokine production are indicators of immunological and hematological abnormalities. The production of cytokines from stimulated leukocytes, fibroblasts, and endothelial cells—primarily interleukin-1 (IL-1), tumor necrosis factor-𝛼 (TNF-𝛼), and IL-6—plays a significant role in the development of a systemic inflammatory response.^[53]^ In our study, we specifically evaluated the release of CRP and IL-6 to assess the differential effects of these various nerve blocks on inflammatory markers. However, to improve the understanding of the efficacy of the anterior iliopsoas space block in elderly hip fracture patients, we suggest that future research include a wider array of inflammatory markers. This will offer a more thorough evaluation of the inflammatory response associated with this nerve block.

Thromboembolic consequences, the impact of anesthetic, and the role of pain medication in the postoperative phase are just a few of the many theories put forth. In addition to lengthy bone fractures and extensive immobility, perioperative anxiety, and surgical techniques might contribute to the high rate of cognitive impairment in orthopedic patients.^[53]^ The reduction in the release of inflammatory markers observed with the anterior iliopsoas space block within 12 hours after surgery may also have implications for the management of other postoperative complications, such as thromboembolic events and wound healing.^[54]^ Inflammation has been recognized as a key factor in the pathophysiology of these complications, and by attenuating the inflammatory response, the anterior iliopsoas space block may contribute to a more favorable postoperative course. For patients suffering from fractures of the hip, FICB provides opioid-sparing analgesia, which lessens pain and lowers the probability of postoperative complications, including delirium and cognitive decline after surgery.^[54]^ In contrast, Jing Dong et al’s study did not find any significant differences in postanesthesia pain scores or intraoperative fentanyl use between the anterior and posterior groups. Nevertheless, the block onset time was longer in the anterior group, with a median difference of 5 minutes compared to the posterior group.^[20]^ The transmuscular quadratus lumborum block combined with the FICB enhances early postoperative recovery.^[4]^ However, studies on FNB and FICB have shown mixed results, with some cases of FICB failing to improve pain relief or function while causing increased quadriceps weakness. Although both blocks are effective for pain control, they pose the risk of motor blockade, particularly affecting quadriceps function, leading to caution in their routine use due to the potential risk of falls.^[6,7,9,55]^ In contrast, the comparison between IPB and FNB in unilateral primary hip arthroplasty highlights that IPB improves quadriceps strength and reduces time to ambulation.^[22]^ The iliopsoas plane is a region where a small injection of local anesthetic can selectively target the sensory branches of the FN innervating the hip joint while preserving the motor function of the quadriceps femoris.^[23]^ IPB targets sensory fibers of the FN and does not interfere with the motor function of the quadriceps femoris.^[23]^ However, the superiority of anterior iliopsoas space block over S-FICB in terms of early postoperative recovery needs to be further investigated to establish the optimal recovery duration for this technique.

Postoperative discomfort perception involves various pain pathways in the peripheral and central nervous systems. While opioids can block nociceptive pain, they do not affect other pathways such as the inflammatory cascade.^[56]^ Multimodal analgesia, which was introduced more than ten years ago, is designed to enhance pain management and reduce the potential for opioid-related adverse effects by using a combination of various types of pain medications to achieve more effective overall pain relief.^[57]^ In our study, we implemented a comprehensive multimodal pain management approach that included preoperative nerve blocks and intraoperative local infiltration analgesia “cocktail.” After surgery, nonsteroidal anti-inflammatory drugs were the preferred option for pain relief, and notably, no patients needed opioids for severe pain. The anterior iliopsoas block has a slight advantage over the S-FICB in managing pain during the recovery phase after hip surgery. Patients receiving this block could have shorter hospital stays and better pain control, particularly during movement, potentially reducing risks like deep vein thrombosis, pulmonary embolism, and postoperative complications. The pain relief provided by the anterior iliopsoas block facilitates early mobilization and reduces inflammatory markers (CRP, IL-6), potentially leading to a faster recovery and improved patient autonomy. However, these findings are not conclusive, and further studies are needed to confirm its long-term benefits on functional outcomes, quality of life, opioid use, postoperative complications, and its potential advantages in clinical practice.

## 5. Limitations

This single-center study has several important limitations that should be considered when interpreting the findings. The relatively small sample size may restrict the generalizability of the results and introduce potential sampling bias. Conducted at a single institution, the outcomes may reflect local practices and specific patient population characteristics, which could limit the external validity of the conclusions. Another significant limitation is the absence of data regarding the duration of analgesia in the both techniques. The study design did not fully account for how the anterior iliopsoas space block and S-FICB impact postoperative pain and analgesic needs. Without a thorough characterization of these block parameters, such as duration of action and the amount of postoperative analgesics required, it is challenging to determine precise differences in pain management after surgery. Furthermore, the study did not evaluate the optimal doses and concentrations of ropivacaine necessary for the effective anterior iliopsoas space block. This lack of data complicates the ability to standardize the technique, compare its efficacy, and assess its potential complications. Further research should focus on identifying the ideal ropivacaine concentration and volume for the anterior iliopsoas space block, as well as examining its influence on postoperative analgesic requirements. Understanding the appropriate dosing and volume of ropivacaine may help standardize the procedure and minimize the risk of postoperative complications, such as inadequate pain relief and LAST.

The narrow focus of the study, which exclusively compared the anterior iliopsoas muscle space block to the S-FICB, without including other regional anesthesia techniques and inflammatory markers for comparison, complicates the ability to draw definitive conclusions regarding the superiority of the anterior iliopsoas block over alternative approaches. The limited data on the other techniques and inflammatory markers restricts the ability to fully assess the comparative pain-relieving effects of anterior space block vs S-FICB. Moreover, the study did not assess the incidence and severity of postoperative delirium, nor did it investigate the long-term consequences of different hip fracture surgeries in this elderly population. The potential impact of the anterior iliopsoas block on the prevention of postoperative cognitive impairment in older hip fracture patients remains unexplored. Furthermore, the comparative effect of these techniques in preventing deep vein thrombosis and other complications associated with hip fracture surgery in this demographic was not evaluated.

To enhance the robustness of future studies, it would be beneficial to incorporate a broader range of regional anesthesia techniques and to include a comprehensive assessment of patient-reported outcomes. Additionally, exploring the biological mechanisms underlying the analgesic effects of the anterior iliopsoas block could provide valuable insights. Future larger, multicenter studies are needed to provide more robust evidence on the comparative effectiveness of the anterior iliopsoas block versus other regional anesthesia techniques. Evaluating the impact of this regional approach on clinically relevant outcomes, such as functional recovery, length of hospital stays, and long-term complications, would further elucidate its potential benefits in the management of elderly hip fracture patients.

## 6. Conclusion

In conclusion, the anterior iliopsoas space block is one of the effective techniques for providing perioperative multimodal analgesia during hip surgery, showing a slight advantage over the S-FICB in elderly patients with hip fractures. However, to fully optimize the effectiveness of the anterior iliopsoas space block, further research is needed to determine the optimal concentration and volume of ropivacaine for this procedure. Such studies are crucial for standardizing the technique and improving patient outcomes in this at-risk population.

## Acknowledgments

The authors have given their Acknowledgments to everyone who made a substantial contribution to this study.

## Author contributions

**Conceptualization:** Peng Ma.

**Data curation:** Juan Zhu, Peng Ma.

**Formal analysis:** Xu Zhang, Peng Jiang.

**Methodology:** Juan Zhu.

**Software:** Xu Zhang, Peng Jiang.

**Writing – original draft:** Emery Niyonkuru, Rui Zeng.

**Writing – review & editing:** Emery Niyonkuru, Rui Zeng.
